# Viroid Pathogenicity: One Process, Many Faces

**DOI:** 10.3390/v1020298

**Published:** 2009-09-10

**Authors:** Robert A. Owens, Rosemarie W. Hammond

**Affiliations:** Molecular Plant Pathology Laboratory, USDA/ARS, Beltsville, MD 20705, USA; E-mail: rose.hammond@ars.usda.gov

**Keywords:** viroid pathogenicity, RNA silencing, disease induction

## Abstract

Despite the non-coding nature of their small RNA genomes, the visible symptoms of viroid infection resemble those associated with many plant virus diseases. Recent evidence indicates that viroid-derived small RNAs acting through host RNA silencing pathways play a key role in viroid pathogenicity. Host responses to viroid infection are complex, involving signaling cascades containing host-encoded protein kinases and crosstalk between hormonal and defense-signaling pathways. Studies of viroid-host interaction in the context of entire biochemical or developmental pathways are just beginning, and many working hypotheses have yet to be critically tested.

## Introduction

Even before the non-coding nature of viroid genomes had been established, how such a small RNA molecule could induce disease was a matter of intense interest. In a 1971 paper comparing the properties of potato spindle tuber “virus” with those of conventional plant viruses and proposing the existence of a new class of pathogens to be known as viroids, T.O. Diener suggested that the genome of *Potato spindle tuber viroid* (PSTVd) might function, not as a messenger RNA, but rather as an abnormal regulatory RNA [1]. Several years later, determination of its complete nucleotide sequence confirmed the non-coding nature of the PSTVd genome [2], and shortly thereafter, comparison of mild and severe PSTVd isolates by RNA fingerprinting revealed that only minor changes in sequence could lead to dramatic effects on symptom expression [3]. As this pioneering era of viroid research drew to a close and the focus began to shift to studies at the molecular level, much effort was devoted to identifying sequence motifs involved in disease induction. Identification of the so-called “pathogenicity domain” in PSTVd and related viroids [4] was soon followed by a proposal that symptom expression might be regulated by the ability of nucleotides within this portion of the molecule to interact with unspecified host components [5]. For many years, the ability of viroids to cause disease was assumed to result from as-yet-unidentified alterations in the normal pattern of RNA-protein interactions.

In 2004, Wang *et al.* [6] reported that expression of a PSTVd-derived hairpin RNA in transgenic tomatoes leads to the appearance of leaf symptoms very similar to those observed in infected plants. Because this hairpin RNA contained less than a full genome equivalent of viroid sequence, these transgenic plants contained no replicating PSTVd. Several earlier studies [7–11] had shown that viroid-infected plants contain small RNAs derived from the invading viroid genome; thus, attention has shifted from RNA-protein interactions to RNA silencing as the primary mediator of viroid pathogenicity. In this review, we draw together key observations from several areas of research on viroid pathogenicity in order to illustrate the history of this area of viroid research and identify promising directions for future studies. In doing so, we depend on several published reviews [e.g., 12–15] as well as the accompanying articles by Ding and Flores elsewhere in this issue to provide the reader with basic information about viroid structure and replication. Here, we focus on studies dealing with i) identification of specific structural elements within viroids that modulate symptom expression, ii) characterization of molecular interactions between these structural elements and specific host components, and iii) determination of the effects of these interactions on host gene expression.

## Identification of structural motifs modulating viroid pathogenicity

**Figure 1.**
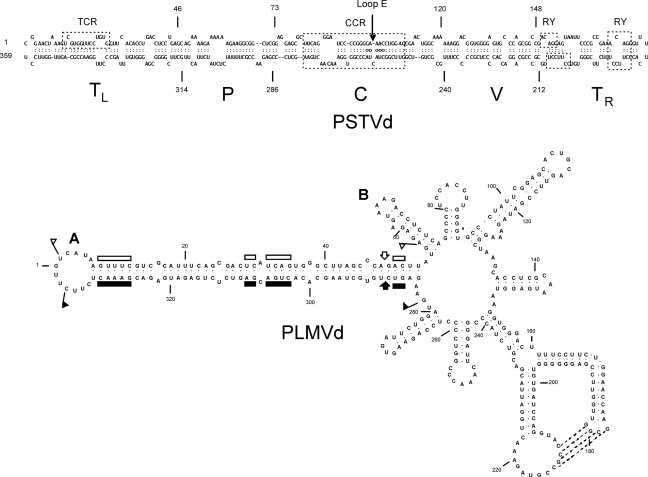
Secondary structures of PSTVd and *Peach latent mosaic viroid* (PLMVd). (**A**) The rod-like secondary structure of PSTVd (intermediate strain) showing the five domains characteristic of members of the family *Pospiviroidae:* the Terminal Left (T_L_), Pathogenicity (P), Central (C), Variable (V), and Terminal Right (T_R_). The Central Conserved Region (CCR) is located within the C domain and contains a UV-sensitive loop E motif with non-canonical base-pairs (denoted by circles). The T_L_ domain of posiviroids contains either a Terminal Conserved Region (TCR) or Terminal Conserved Hairpin (location not shown). The T_R_ domain may contain 1–2 copies of a protein-binding RY motif [16]. (**B**) The branched secondary structure of PLMVd, a member of the ribozyme-containing family *Avsunviroidae*. Boundaries of the plus and minus strand self-cleavage domains are indicated by flags, nucleotides conserved in most natural hammerhead structures by bars, and the self-cleavage sites by arrows. Filled and open symbols refer to plus and minus polarities, respectively. Nucleotides involved in a pseudoknot supported by chemical probing are indicated by broken lines. Co-variation analysis suggests that a second pseudoknot may exist between loops A (location of the short insertion responsible for peach calico) and B.

Determination of the complete nucleotide sequence of PSTVd by Gross and colleagues [2] was rapidly followed by several similar reports involving other viroids and sequence variants. In 1985, Keese and Symons [4] used this information to propose that PSTVd and related viroids contain five structural and functional domains. As shown in Figure 1, these domains include i) a conserved central domain [C] capable of forming two (or more) alternative structures that may regulate the replication cycle, ii) a domain associated with pathogenicity [P], iii) a domain exhibiting high sequence variability [V], and iv) two terminal domains that are interchangeable between viroids [T_L_ and T_R_]. In addition to focusing attention on the possible role of a defined pathogenicity domain in regulating the disease process, this seminal paper also drew attention to the probable role of RNA recombination in viroid evolution. More than 1700 viroid sequences are now available in the Subviral RNA Database (http://subviral.med.uottawa.ca/).

**Figure 2.**
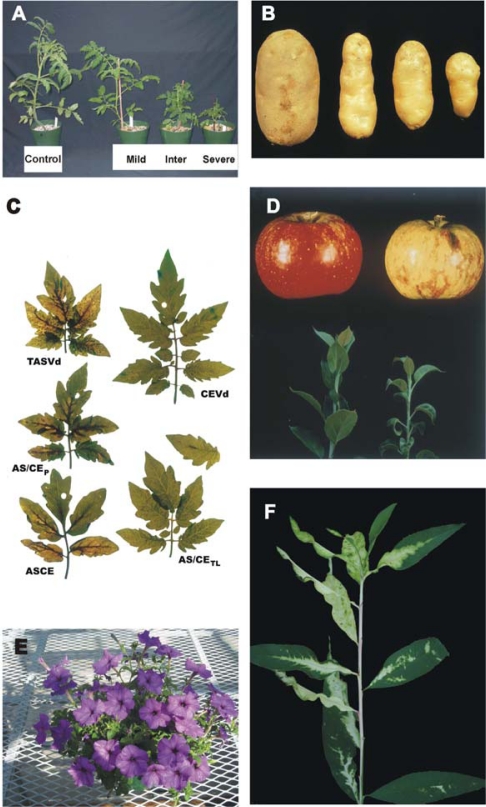
Symptoms associated with viroid infection. (**A,C**) Infection of sensitive tomato cultivars with PSTVd or related viroids like CEVd and TASVd leads to stunting, epinasty (a downward curling of the leaves), and veinal necrosis. Note the differences in symptom severity associated with different strains of PSTVd (**A**) or different viroids (**C**). (**B)** Symptoms of PSTVd in its natural host (potato); the control tuber on the left is from a healthy plant. (**D**) Fruit from viroid-infected woody hosts like apple or plum may exhibit abnormal pigmentation; *i.e.*, “color break”. Left, healthy apple; right, *Apple scar skin viroid* (ASSVd)-infected apple. (**F**) Infection of peach by certain variants of PLMVd leads to extreme chlorosis and loss of chlorophyll from large portions of the leaves (courtesy of Francesco Di Serio). (**E**) Many viroid-infected plants (especially herbaceous ornamentals like this *Tomato chlorotic dwarf viroid* (TCDVd)-infected petunia) may show no visible symptoms. Vegetative propagation of latently infected plant material dramatically increases the number of viroid-infected plants, thereby increasing the opportunity for accidental transfer (*i.e.*, “escape”) to other sensitive species growing nearby.

Many of the studies subsequently carried out to identify structural motifs modulating viroid pathogenicity have focused on PSTVd and related viroids. PSTVd and closely-related viroids like *Citrus exocortis viroid* (CEVd) or *Tomato apical stunt viroid* (TASVd) all infect tomato, and when bioassays are carried out using certain sensitive cultivars (e.g. Rutgers), infected plants exhibit a characteristic range of symptoms (see Figure 2). Tomato bioassays are both rapid and convenient – requiring only 4–6 weeks for completion compared to the months or more required for assays involving their natural hosts. As discussed below, the results of many (but not all) of these studies point to small RNAs and RNA silencing as key intermediaries in the disease process.

Two lines of evidence indicate that nucleotides within in the P domain play an important role in PSTVd symptom induction: First, the fact that only 1–2 changes in this portion of molecule are sufficient to dramatically alter symptom expression in tomato [3]; second, the association of a larger number of sequence changes restricted mainly to the P and V domains of CEVd with similar effects [17]. For several naturally-occurring isolates of PSTVd, thermodynamic calculations indicate that virulence is correlated with the instability of a single structural motif; *i.e.*, the so-called “virulence modulating region” within the P domain. Based on this correlation, Schnölzer *et al.* [5] proposed that PSTVd virulence is determined by the ability of nucleotides within this VM region to interact with one or more unidentified host factors.

The first indication that the molecular mechanism(s) underlying viroid pathogenicity might not be quite so simple came soon thereafter when analysis of CEVd isolates failed to reveal a similar correlation [17]. To investigate the possible contributions of other structural domains to pathogenicity, Sano and colleagues [18] constructed a series of interspecific chimeras between CEVd and TASVd and examined infected plants for differences in various features of symptom development; e.g., stunting, veinal necrosis, and epinasty. The individual contributions of the T_L_ (terminal left) and P domains to symptom induction were not completely separable from effects on viroid titer, but sequence differences in the T_L_ domain appeared to have a greater effect on symptom severity than changes in the P domain. Three discrete regions of sequence and/or structural variability were identified that may correspond to these pathogenicity determinants.

Characterization of naturally-occurring variants of several other viroids -- *Hop stunt viroid* (HSVd), *Coconut cadang-cadang viroid* (CCCVd), *Avocado sunblotch viroid* (ASBVd), *Chrysanthemum chlorotic mottle viroid* (CChMVd), and PLMVd – has identified still more pathogenicity determinants. For example, HSVd variants isolated from citrus trees exhibiting symptoms of cachexia contain a characteristic cluster of six specific changes in the V domain [19,20]. Cachexia and xyloporosis are graft-transmissible diseases of citrus that are characterized by the development of severe gumming, discoloration, and stem-pitting symptoms in specific indicator hosts. CCCVd is the smallest known pospiviroid (*i.e.*, 246 nt) and causes a lethal disease of coconut palm in the Philippines. Artificial passage of CCCVd led to the appearance of a severe lamina-depleting symptom (“brooming”) that was associated with sequence changes at three sites in the P and C domains [21]. Changes were observed at one or two of these sites, but not at all three sites simultaneously. Interestingly, certain of these changes were located just outside the loop E motif of CCCVd, a motif shown by the work of Ding and colleagues to play an important role in PSTVd replication and pathogenicity (see below).

ASBVd is a member of the *Avsunviroidae*, a second family of ribozyme-containing viroids that replicate in the chloroplast, and its rod-like secondary structure does not contain the five structural/functional domains found in PSTVd and related viroids. Characterization of ASBVd variants associated with bleached, variegated, or symptomless leaf tissue suggest a transition in sunblotch disease from a severe acute to a persistent mild form of infection [22]. During this transition, sequence changes accumulating in the right terminal loop may lead to a more open structure of this portion of the molecule, potentially altering its ability to bind RNA polymerase. Similar results were reported by Schnell *et al.* [23]. PLMVd and CChMVd are two other ribozyme-containing viroids whose branched secondary structures differ dramatically from the overall rod-like structure of ASBVd (see Figure 2). Comparisons of symptomatic and asymptomatic strains of CChMVd have shown that sequence changes in a single hairpin loop are sufficient to convert a symptomatic strain to an asymptomatic one. The substitution involved (*i.e.*, UUUC→GAAA) creates a highly stable GNRA tetraloop that, in addition to abolishing symptom expression, also reduces overall fitness [24, 25]. Peach trees infected with certain isolates of PLMVd exhibit an extreme chlorosis covering most of the leaf area that is known as “peach calico”. Sequence analysis of full-length cDNAs derived from such isolates revealed two groups of variants, one containing a 12–13 nt insertion in the hairpin loop that caps the hammerhead stem (see Figure 2). Using site-directed mutagenesis and bioassays on a sensitive indicator host, Malfitano *et al.* [26] were able to show that i) variants lacking this insertion replicate without eliciting symptoms and ii) that this insertion can sporadically emerge *de novo*.

How these proposed pathogenicity determinants actually influence the disease process remains to be determined. In many cases, the most likely mechanism appears to involve synthesis of viroid-related siRNAs and RNA silencing. Highly stable GNRA tetraloops like the one found in CChMVd are known for their ability to act as protein-binding sites [27], however, and the presence of such a structure in small siRNAs would tend to interfere with their ability to base-pair with a potential mRNA target. A second example of a viroid pathogenicity determinant likely to act in the context of the genomic RNA is the loop E motif located in the central domain of PSTVd.

Loop E motifs are a common feature of many cellular RNAs, where they help to organize multihelix loops and other elements of tertiary structure [28]. The presence of such a highly-structured, UV-sensitive motif loop in the central domain of PSTVd and related viroids was first recognized in 1985 [29], but its possible function(s) remained obscure until Wassenegger and colleagues [30] reported that replacement of the C residue normally found at PSTVd position 259 with U dramatically increased the viroid’s ability to replicate in tobacco. Several years later, Qi and Ding [31] reported that a U/A change at nearby position 257 results in a very unusual “flat top” phenotype. This mutation did not enhance the rate of PSTVd replication/accumulation, and its effects were independent of symptom determinants located in the P domain. The loop E motif of PSTVd was recently subjected to a very comprehensive mutational analysis [32] whose results were in remarkable agreement with structural predictions derived from earlier studies of loop E motifs in other RNAs. Like GNRA tetraloops, loop E motifs often act as sites for RNA-protein interaction [33]; furthermore, the sequences which interact to form the PSTVd loop E motif are widely separated in the genomic RNA. No cellular proteins have yet been shown to interact with the loop E motif of PSTVd, but among the more intriguing candidates is a tobacco RIP (ribosome-inactivating protein)-like protein with dual enzymatic activity [34]. The conserved hairpin loop in mammalian rRNA recognized by the cytotoxic proteins α-sarcin and ricin contains a loop E motif very similar to the one found in PSTVd.

## Viroid-protein interaction as a potential trigger for symptom induction

Several groups have examined the ability of viroids to interact with cellular proteins. In one early study, Wolff *et al.* [35] used one-dimensional SDS-PAGE and Northwestern analysis to demonstrate the ability of circular PSTVd to interact with several different tomato proteins; namely, all four histones plus two larger nuclear proteins approximately 31 kDa and 41 kDa in size. Follow-up studies by Klaff *et al.* [36] compared the ability of tomato nuclear proteins to interact *in vitro* with either linear PSTVd RNA transcripts or circular PSTVd. Other experiments used a combination of UV cross-linking followed by RNase digestion to identify several viroid-protein complexes in isolated nuclei. A 43 kDa protein could be isolated from the cellular complexes by RNase digestion, but the identity/cellular function of this protein was not determined. Yet another study from the Riesner laboratory demonstrated the ability of purified wheat germ RNA polymerase II to interact with both PSTVd terminal loops [37], the first time that a viroid-protein interaction had been mapped to a specific structural motif. Relatively recently, PSTVd (−)strand RNA synthesis was shown to initiate in the left terminal loop of the circular (+)strand template [38].

Over the years, several additional viroid-binding proteins have been characterized. A series of studies from the Tabler-Tsagris laboratory [e.g.,^16^,^39^] have characterized a bromodomain-containing tomato protein known as VIRP1 that specifically binds to an AGG/CCUUC motif found in the right terminal domain of PSTVd (see Figure 1). This interaction with VIRP1 appears to play an important role in the long distance movement of PSTVd and other pospiviroids in the host vascular system. A second cellular protein that appears to be involved in long distance movement of viroids is the phloem lectin known as PP2, a moderately basic 49-kDa dimeric protein that preferentially binds N-acetylglucosamine oligomers and is one of the two most abundant proteins in phloem exudate. Cucumber PP2 interacts with HSVd both *in vitro* [40,41] and *in vivo* [42]; furthermore, characterization of the gene encoding cucumber PP2 revealed the presence of a potential dsRNA-binding motif. Because the interaction between HSVd and PP2 appears rather non-specific (*i.e.*, PP2 can bind a number of other RNA molecules), it would seem to be an unlikely trigger for pathogenesis. Finally, the genome of ASBVd has been shown to interact with two small chloroplast RNA binding proteins encoded by the nuclear genome of its host [43]. Binding of PARBP33 and PARBP35 to multimeric ASBVd RNA transcripts stimulates hammerhead ribozyme-mediated self-cleavage *in vitro*, an interaction that seems more likely to be involved in replication rather than disease induction.

Signaling cascades involving at least two host-encoded protein kinases appear to play a role in modulating viroid pathogenicity. PSTVd infection of tomato leads to the autophosphorylation of a plant-encoded 68 kDa analog of PKR, the mammalian double-stranded RNA-dependent protein kinase implicated in the regulation of animal RNA virus replication [44,45]; furthermore, incubation of purified mammalian PKR with RNA transcripts derived from a severe strain of PSTVd resulted in 10-fold greater activation than incubation of the kinase with transcripts derived from a mild strain [46]. Although these observations strongly suggest a direct interaction between PKR and a sequence motif in the pathogenicity domain of PSTVd, direct evidence for such an interaction is lacking. To date, all attempts to extend these studies by cloning the mRNA and/or gene encoding tomato PKR have been unsuccessful. Infection of tomato seedlings with either the intermediate or a severe strain of PSTVd also results in the transcriptional activation of a second, 55 kDa protein kinase known as PKV (*i.e.*, protein **k**inase **v**iroid-induced; 47). Sequence analysis of the gene encoding PKV (*pkv*) revealed significant homologies to cyclic nucleotide-dependent protein kinases, and the ability of recombinant PKV protein to autophosphorylate on serine and tyrosine residues *in vitro* suggests that it belongs to the class of dual-specificity protein kinases. Further analysis of PKV revealed that it is a novel member of the AGC VIIIa protein kinase superfamily [48], however, little is known about the role of AGC VIIIa kinases in plants. Within subgroup AGC VIIIa, only PINOID [49] and Adi3 [50] have been genetically characterized, and have been shown to play fundamental roles in auxin signaling and plant cell death, respectively. As discussed in more detail below, recent studies have shown that PKV may play a role in gibberellic acid (GA) signaling. While infection by certain strains of PSTVd results in transcriptional activation of *pkv*, it is not known if PSTVd directly binds to the PKV protein to trigger pathogenesis.

## Host responses to viroid infection

The visible symptoms of viroid infection resemble those associated with many plant virus diseases and include stunting, epinasty, leaf distortion, vein discoloration or necrosis, vein clearing, chlorotic or necrotic spots, mottling and necrosis of leaves, and (rarely) death of the entire plant. As discussed by Diener [51], this similarity in symptomology suggests that the host metabolic pathways affected by viroid and virus infection are very similar. At the cellular level, viroid infection has been associated with disruption/proliferation of the plasma membrane as well as various abnormalities affecting the chloroplast and cell wall. Members of the *Avsunviroidae* including ASBVd or PLMVd replicate in the chloroplast; thus, their ability to disrupt chloroplast structure, thereby leading to leaf chlorosis and bleaching, is not unexpected [22,26]. Effects on chloroplast metabolism may be a more common consequence of viroid infection than generally recognized, however, because PSTVd and several related viroids replicating in the nucleus have similar effects.

At the molecular level, the effects of viroid infection on host gene expression have been examined at both the transcriptional and post-transcriptional levels. Several studies have described the effect of viroid infection on the transcription levels of individual genes. For example, viroid infection of tomato results in increased transcription of stress-induced and defense-related genes, including those encoding pathogenesis related (PR) proteins, PR1a and PR1b, and β-1, 3-glucanases, among others; in this respect, the plant response to viroid infection is similar to the response to bacterial, fungal, and/or viral infection [52–54]. In addition to transcriptional activation of host genes, PSTVd infection of tomato also results in reduced transcription of the *LeExp2* expansin gene, suggesting that stunting results from restricted cell expansion [31]. Growth reduction in citrus caused by infection with *Citrus exocortis viroid* (CEVd) was correlated with reduced levels of gibberellin 20-oxidase mRNA [55].

As described earlier, PSTVd infection of tomato results in the transcriptional activation of the serine-threonine protein kinase-encoding *pkv* gene. Further studies focused on the biological role of PKV in plant development revealed that over-expression of PKV in tobacco resulted in dwarfing and reduced root formation, similar to symptoms of PSTVd infection in tomato [48]. Hormone supplementation and gene expression analyses suggested that gibberellic acid biosynthetic and/or signaling pathways are regulated by PKV, resulting in lower levels of active GAs and a resulting dwarf phenotype. While both positive and negative signaling components in GA signal transduction pathways have been characterized, and include the DELLA proteins and bZIP transcriptional activators/repressors [56,57], their role in biochemical pathways responsible for the dwarfing phenotype in viroid-infected plants is not yet understood.

Host responses to viroid infection are complex and may involve crosstalk between hormonal and defense-signaling pathways. Unlike viroid-infected tomato plants, transcription of PR genes is not induced in tobacco plants that over-express PKV, suggesting that *pkv* downstream signaling is not through salicylic acid- or jasmonic acid-dependent pathways. The putative promoter region of *pkv* contains G- (CACGTG) and H-box (GGTAGG) *cis* elements [47] which are known to interact with, and to be transcriptionally activated by, bZIP transcriptional factors. These *cis* elements are thought to be responsible for early responses to pathogen attack [58], but they also function in the regulation of genes by developmental stimuli [59]. The genes encoding PKV and the PR proteins may be transcriptionally activated by the same bZIP transcription factors but, in tomato, their gene products appear to diverge into separate signaling pathways. There may also be spatial and temporal regulation of the host response to viroid infection. A more global analysis of gene expression may provide answers to these questions.

Only two published studies [60,61] have examined the effects of viroid infection at the transcriptional level. Using a collection of 1,156 partial tomato cDNA clones obtained by PCR-based cDNA library subtraction, Itaya *et al.* [60] compared changes in gene expression induced by mild and severe strains of PSTVd with those caused by *Tobacco mosaic virus* (TMV). Of 55 genes whose expression levels were altered by viroid infection, approximately one-third were also affected by TMV. These genes encode products involved in defense/stress responses, cell wall structure, chloroplast function, and protein metabolism. One of five up-regulated genes detected by differential display analysis of Etrog citron leaves infected by *Citrus viroid III* encodes a calmodulin-related suppressor of RNA silencing [61]. Clearly, viroid infection triggers a complex series of host responses, but additional studies using larger arrays and improved technologies (e.g., high through-put cloning and sequencing) are required to identify individual signaling pathways involved. A recent analysis of changes in Arabidopsis gene expression associated with geminivirus infection, for example, uncovered 5,365 genes that are differentially expressed 12 dpi [62]. The Affymetrix ATH1 GeneChip used for this analysis contains probes for approximately 24,000 genes.

## Role(s) of RNA silencing in viroid pathogenesis

Over the last decade, small (20–30 nt) RNAs have emerged as critical regulators of eukaryotic gene expression, and many excellent reviews summarizing progress in characterizing the diverse pathways and regulatory mechanisms involved have appeared [e.g., 63–67]. The first evidence that RNA silencing might play a key role in modulating viroid-host interaction appeared in 1994 when Wassenegger *et al.* [68] reported that certain PSTVd cDNAs became specifically methylated following introduction into the tobacco genome via *Agrobacterium*-mediated leaf-disc transformation. The involvement of small RNAs in this process was not yet apparent, but one eventual link to RNA silencing was clear; *i.e.*, methylation was dependent upon viroid replication, and non-infectious PSTVd cDNAs remained unmethylated. Several years later as interest in RNA silencing as a key regulator of eukaryotic gene expression was rapidly increasing, several groups reported the presence of small, viroid-related RNAs in plants infected via conventional routes of inoculation. A recent review summarizing the evidence that RNA silencing plays a major role in viroid pathogenesis and evolution [69] concludes with a model that links synthesis of *trans*-acting small interfering RNA (ta-siRNA) with viroid replication and pathogenesis. We focus here on several gaps in this evidence where additional studies appear most promising.

As shown in Table 1, the two currently recognized broad classes of plant small RNAs - microRNAs (miRNAs) and small interfering (si)RNAs – differ in several important respects. In Arabidopsis, four different DICER-LIKE (DCL) activities and a variety of other proteins (including as many as six RNA-dependent RNA polymerases) are involved in small RNA synthesis. Cleavage of the respective precursor molecules releases different sized small RNAs, and genetic analysis has revealed considerable overlap/redundancy in DCL activity. Viroid-derived (vd)siRNAs have been detected in plants infected by several different viroids, some replicating in the nucleus (PSTVd, CEVd, HLVd, and HSVd) and others in the chloroplast (*i.e.*, ASBVd). There is no consistent relationship between (vd)siRNA concentration and symptom severity, but development of visible symptoms in PSTVd-infected plants is accompanied by a shift from 21–22 to larger 24-nt siRNAs [70].

Several lines of evidence suggest that a significant proportion of (PSTVd)siRNA is derived from the genomic RNA. Incubation of PSTVd or PLMVd RNA transcripts with DCR-containing extracts *in vitro* results in the release of ∼21-nt small RNAs [71,72]. Sequence analysis of PSTVd [70,71] and CEVd [73] siRNAs recovered from infected tomato plants indicates that a large majority of 21-nt (vd)siRNAs originate from “hotspots” on the genomic RNA that do not include the pathogenicity domain (a portion of the genome known to play a key role in modulating PSTVd symptom expression). To date, the amplification and cloning methodologies used for (vd)sirRNA analysis have been 5′-ligation-dependent; thus, molecules with modified 5′-termini may have been severely under-represented. While the distribution of PSTVd-related small RNAs isolated from transgenic tomatoes expressing a less-than-full-length PSTVd hairpin RNA appears very similar to that observed with infected plants [74], a large proportion of the siRNAs generated by incubation of dsPSTVd RNA with recombinant Dicer *in vitro* were derived from the pathogenicity domain [71]. New and improved “deep sequencing” strategies will undoubtedly lead to significant changes in current ideas regarding (vd)siRNA biogenesis.

How (vd)siRNAs induce disease in susceptible hosts is not yet clear. GFP-reporter genes fused to partial sequence of PSTVd are silenced when expressed in PSTVd-infected plants [71,76] indicating that (PSTVd)siRNAs can directly target host mRNAs for RISC-mediated degradation. Interestingly, PSTVd replication itself appears resistant to RNA silencing (71; for an opposing view, see 77). Viroid siRNAs might also act indirectly by altering levels of host siRNA metabolism. For example, miRNA-based regulation is integral to pathways controlling plant growth and development, and miRNA biogenesis is known to be affected by both abiotic and biotic stresses [64]. Certain symptoms such as epinasty and rugosity commonly associated with viroid infection reflect changes in leaf developmental patterns, so this question needs to be addressed. In the case of CEVd-infected tomato, however, the answer appears to be “No”. Expression levels of four miRNAs, transcripts encoding the DCL1 and AGO1 activities required for miRNA synthesis, as well as two miRNAs regulating DCL1 and AGO1 expression were not affected by CEVd infection [73].

**Figure 3.**
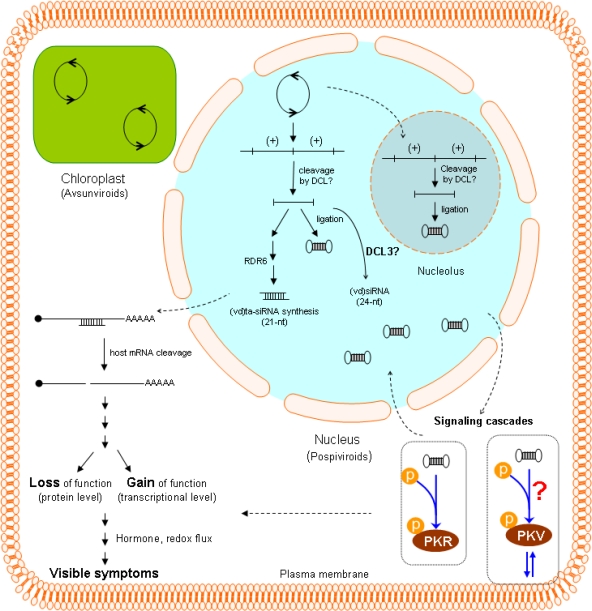
Schematic overview of viroid pathogenicity illustrating both direct and indirect interaction between the viroid genome and host cell. As discussed by Gomez *et al.* [69], RNA silencing mediated by (vd)ta-siRNAs appears likely to play a major role in disease induction by viroids like PSTVd or HSVd that replicate in the nucleus. The resulting cleavage of host mRNAs could lead to either loss or gain of function at protein level. PSTVd (and presumably other viroid RNAs) also activates at least two protein kinases, one of which (PKV) may be associated with the plasma membrane. Activation of the signaling cascades containing these kinases would then lead to perturbations in plant defense and hormone signaling pathways. Very little is known about siRNA synthesis associated with viroid replication in the chloroplast. Viroid replication in both the nucleus and cytoplasm proceeds via a rolling circle mechanism (indicated by open circles with opposing arrows). For viroids like PSTVd that replicate in the nucleus, various stages of replication appear to be localized in either the nucleoplasm or the nucleolus [75].

A recent study from the Pallás laboratory [78] showing that symptom development in transgenic *Nicotiana benthamiana* plants that express a dimeric form of HSVd is dependent upon expression of RNA-dependent RNA polymerase 6 (RDR6) adds yet another level of complexity to this situation. In non-transgenic plants, symptom development can be suppressed by growth at low temperatures (*i.e.*, 14° C), temperatures at which RNA silencing is also reduced [79]. As discussed by Gómez *et al.* [69], RDR6 plays a key role in tasiRNA synthesis, and the template for (HSVd)ta-siRNA synthesis may be the monomeric linear progeny released when DCL cleaves multimeric HSVd (+)strand RNA within a so-called “trihelical region” formed by base-pairing between two copies of an imperfect inverted repeat in the upper portion of the C domain (80; see also the accompanying review by Flores). In contrast to the intramolecular nature of this HSVd cleavage reaction, cleavage of cellular (pre)ta-siRNAs requires miRNA binding. Figure 3 shows some of the pathways by which (vd)siRNAs may influence host gene expression, thereby resulting in visible disease.

## Potential targets of viroid-mediated RNA silencing

Microarray analysis and other forms of transcriptome profiling allow the study of host-pathogen interactions in the context of entire biochemical or developmental pathways. As discussed in several recent reviews [e.g., 81–83], virus infection has been shown to i) induce a variety of plant defense and stress responses and ii) down-regulate other genes with potential roles in plant growth and development. Processes affected include hormone and developmental signaling, transport in the vascular system, cell reprogramming, RNA silencing, and protein modification/relocation/degradation. Salicylic acid (SA)-mediated signaling plays a key role in compatible interactions between RNA viruses and their plant hosts [82]; for small DNA viruses like *Cabbage leaf curl virus* whose genomes are dependent on the DNA replication machinery of the host, infection results in altered expression of numerous cell cycle-associated genes [62]. Similarities in the visible symptoms induced suggest that plants respond to the presence of viroids and viruses in a very similar fashion, but this hypothesis has yet to be critically tested.

At the present time, PSTVd and its experimental host, tomato, provide the most suitable viroid/host combination for transcriptome profiling studies. Arabidopsis would normally be the host of choice for such studies, but unknown factors severely restrict viroid replication and movement in this model plant [84]. Sequencing of the tomato genome is now almost 50% complete, and several critical bioinformatics tools have recently been added to the Tomato Functional Genomics Database (http://ted.bti.cornell.edu/); *i.e.*, sRNA and miRNA databases as well as web-based tools to predict sRNA:mRNA interactions and identify changed pathways and biological processes from gene/protein expression and metabolite profile datasets. Our laboratories are currently comparing the changes in transcriptome profiles associated with PSTVd infection in two different tomato cultivars with those observed in transgenic plants expressing a noninfectious PSTVd hairpin RNA [6]; analysis of results from preliminary studies is now complete, and results from the entire study should be available soon. Among the questions that can be addressed using these new bioinformatics tools: What proportion of down-regulated genes contain potential binding site(s) for (PSTVd)siRNAs? Is there evidence for mRNA cleavage at the predicted binding sites?

## Beyond genes and pathways…

PSTVd (and presumably other pospiviroids) is known to accumulate in the nucleolus of infected cells where its presence is associated with the redistribution of small nucleolar RNA U3 [75,85]. The nucleolus is a dynamic subnuclear structure with roles in ribosome biogenesis, mediation of cell-stress responses and regulation of cell growth, and its structure and proteome are constantly changing in response to metabolic conditions. Many RNA and DNA viruses interact with the nucleolus to usurp host-cell functions and recruit nucleolar proteins to facilitate virus replication [86], and transcriptome profiling may reveal whether or not viroid infection has similar effects. Datasets for geminiviruses [62] and a plant rhabdovirus that replicates in the nucleus [87] are available for comparison. It is not difficult to imagine how this still poorly-understood process could disrupt normal transport of host proteins and RNAs – with far-reaching consequences on both regulatory and metabolic pathways.

Finally, recent progress in dissecting the mechanisms through which virus-host interactions affect host physiology [88] suggest that it may be time to take a fresh look at the metabolic changes associated with viroid infection. For example, the same phenylpropanoid-derived isoflavonoids that serve as primary defense compounds and key signaling molecules mediating plant-microbe interactions [89] also act as anti-oxidants that buffer plant cells against changes in redox status. Redox potential appears to have been used for signal transduction from very early evolutionary times, and changes in cellular redox status have been shown, in animals, to regulate signal transduction and many other important physiological processes [90]. Senescence and disease-related responses involving programmed cell death have long been known to increase the rate and alter the pattern of phenylpropanoid biosynthesis [91]. Because secondary metabolites like the products of phenylpropanoid metabolism are transported across membranes by specific carrier proteins, it may be possible to use changes in apoplastic phenolics to monitor metabolic changes associated with viroid infection inside host cells. Moreover, crosstalk between salicylic acid/jasmonic acid plant defense signaling pathways and hormone signaling pathways [92] likely plays a role in viroid pathogenesis and presents challenges in unraveling the complex interactions leading to symptom formation.

## Figures and Tables

**Table 1.** Major pathways of plant small RNA synthesis.

	miRNA synthesis	siRNA synthesis		

Dicer Precursor	DCL1 Pri-miRNA	DCL2	DCL3	DCL4
Primary product (size)	miRNA (21-nt)	siRNA (22-nt)	siRNA (24-nt)	siRNA (21-nt)
Downstream events	Transcript cleavage		DNA and histone methylation	
Additional factors involved	RDR6 DCL4 > DCL2		RDR2 RNA pol IV	
Secondary product	Ta-siRNAs			
